# DNA methylation-based analysis reveals accelerated epigenetic aging in giant cell-enriched adult-type glioblastoma

**DOI:** 10.1186/s13148-024-01793-w

**Published:** 2024-12-11

**Authors:** Pinar Cakmak, Philipp Jurmeister, Iris Divé, Pia S. Zeiner, Joachim P. Steinbach, Tim R. Fenton, Karl H. Plate, Marcus Czabanka, Patrick N. Harter, Katharina J. Weber

**Affiliations:** 1 Goethe University Frankfurt, University Hospital, Neurological Institute (Edinger Institute), Frankfurt, Germany; 2 Goethe University Frankfurt, Frankfurt Cancer Institute (FCI), Frankfurt, Germany; 3 Ludwig Maximilians University Munich, University Hospital, Institute of Pathology, Munich, Germany; 4 German Cancer Consortium (DKTK), Partner Site Munich, a partnership between German Cancer Research Center (DKFZ) and University/University Hospital, Ludwig Maximilians University Munich, Munich, Germany; 5 Goethe University Frankfurt, University Hospital, Dr. Senckenberg Institute of Neurooncology, Frankfurt, Germany; 6 German Cancer Consortium (DKTK), Partner Site Frankfurt, and German Cancer Research Center (DKFZ), Heidelberg, Germany; 7 Goethe University Frankfurt, University Hospital, University Cancer Center (UCT), Frankfurt, Germany; 8 Goethe University Frankfurt, University Hospital, Department of Neurology, Frankfurt, Germany; 9 Somers Cancer Research, Southampton General Hospital, Southampton, UK; 10 Goethe University Frankfurt, University Hospital, Department of Neurosurgery, Frankfurt, Germany; 11 Ludwig Maximilians University Munich, University Hospital, Center for Neuropathology and Prion Research, Munich, Germany

**Keywords:** Giant cell glioblastoma, Epigenetic aging, DNA methylation age acceleration, Pediatric-type high-grade glioma

## Abstract

**Background:**

Giant cell (gc)-enriched glioblastoma (gcGB) represents a distinct histological variant of isocitrate dehydrogenase wild-type adult-type glioblastoma with notable enlarged mono- or multinuclear tumor cells. While some studies suggest a survival advantage for gcGB patients, the underlying causes remain elusive. GcGBs are associated with TP53 mutations, and gcs were shown to accumulate DNA double-strand breaks and show deficient mitosis, potentially triggering cellular senescence programs. Epigenetic clocks have emerged as valuable tools for assessing tumor-induced age acceleration (DNAMethAgeAcc), which has lately proved itself as prognostic biomarker in glioblastoma. Our study aimed to comprehensively analyze the methylome and key metabolic proteins of gcGBs, hypothesizing that they undergo cellular aging programs compared to non-gcGBs.

**Results:**

A total of 310 epigenetically classified GBs, including 26 gcGBs, and nine adults with malignant gliomas allocating to pediatric high-grade glioma molecular subclasses (summarized as “pediatric GB”) were included. DNAMethAgeAcc was computed by subtraction of chronological patient ages from DNA methylome-derived age estimations and its increase was associated with better survival within gcGB and non-gcGB. GcGBs were significantly more often allocated to the subgroup with increased DNAMethAgeAcc and demonstrated the highest DNAMethAgeAcc. Hypothetical senescence/aging-induced changes of the tumor microenvironment were addressed by tumor deconvolution, which was able to identify a cluster enriched for tumors with increased DNAMethAgeAcc. Key metabolic protein expression did not differ between gcGB and non-gcGB and tumor with versus without increased DNAMethAgeAcc but for elevated levels of one single mitochondrial marker, anti-mitochondrial protein MT-C02, in gcGBs.

**Conclusions:**

With its sped-up epigenetic aging, gcGB presented as the epigenetic oldest GB variant in our cohort. Whereas the correlation between accelerated tumor-intrinsic epigenetic aging and cellular senescence in gcGB stays elusive, fostering epigenetic aging programs in GB might be of interest for future exploration of alternative treatment options in GB patients.

**Supplementary Information:**

The online version contains supplementary material available at 10.1186/s13148-024-01793-w.

## Background

Glioblastoma, IDH wild-type CNS WHO grade 4 is the most frequent malignant primary brain tumor in adults [1]. The recent years’ comprehensive efforts in an epigenetically based brain tumor classification by use of DNA methylation profiling shaped our knowledge about the epigenetic variety of malignant gliomas[2, 3]. To this end and among histone 3/IDH wild-type tumors, DNA methylation analysis frames the spectrum of histologically higher-grade gliomas encompassing high-grade gliomas in adults with various subtypes beyond classic GB, and pediatric-type high-grade gliomas[4]. The latter was shown to rather cluster with other pediatric neoplasms like medulloblastoma or the former PNET group of CNS tumors than adult GB in genome-wide molecular analyses which let its first describers draw tentative conclusions about a dependency from chronological age[5, 6]. Adult GB displays morphological variants, which are listed as subtypes in the current CNS WHO tumor classification but are allotted within the same DNA methylation class. GcGB is regarded as one rare subtype with an incidence of 1–5% among GB and a 1.4- to 1.5-fold male predominance[7–9]. It is characterized by markedly big and pleomorphic, mono- or multinucleated nuclei of bizarre shape[4]. With a median age of 51 versus 62 years at first diagnosis, gcGB was found to occur about a decade earlier than GB without enrichment for giant cells (non-gcGB)[7]. Patients suffering from gcGB were shown to have a better overall survival than those with non-gcGBs in some studies[7]. In part, this finding has been attributed to younger patient age at presentation at clinics favoring higher extents of surgical tumor resection[7]. On the contrary, pointing toward a more beneficial tumor-intrinsic factor, gcGB was inclined to a less infiltrative growth pattern and the presence of gc within diffuse glioma portended an overall survival benefit independent of extent of resection and patient age[7, 10]. Still so far, the reasons for survival advantage of gcGB patients are not completely understood.

Data on comprehensive DNA methylation analyses in gcGB are scarce. In a study from 2018, DNA methylation patterns of gcGB overlapped with those of non-gcGB, thus not justifying a methylation-based subtyping of gcGB[11]. From a genetic perspective however, gcGB holds some specific alterations. Therefore, gcGB was found to harbor higher frequencies of altered *RB1*, *NF1*, *TP53* and *ATRX* genes as well as genes related with mismatch repair deficiency in comparison with non-gcGB, whereas *EGFR* alteration occurred less frequently[12]. Hypermutation was described in up to 41% of gcGBs, and an average of 76% of tumor cells readily showed gammaH2AX immunoreactivity indicative of an accumulation of DNA double strand breaks [10, 12]. Some studies defined a dysfunction of the mitotic serine/threonine kinase Aurora B as the molecular basis of the defective chromosome separation and cytokinesis in synergy with unhindered cell cycling in a p53-compromised context leading to the formation of gcs[13–16].

One of the cell cycle checkpoint p53’s main functions is the blockage of cell proliferation upon detection of DNA damage. One way of preventing deleterious cells from cell cycling is the initiation of cellular senescence programs. Cellular senescence is orchestrated by p53 together with p21 as well as by Rb/CDKN2A and is understood as a state of metabolic and paracrine activity but exit from cell division cycles due to irresponsiveness to mitogene signaling. Among many others shortening of telomeres, expression of gammaH2AX and p21, upregulation of CDKN2A/B, functional impairment of mitochondria and epigenetic alterations commonly leading to a loosening of chromatin compaction have been described as hallmarks associated with cellular senescence[17]. Nevertheless, senescent cells can potentially re-enter into cell cycling when oncogenes are expressed additionally to encroachment of p53, thus leading to the multiplication of mutant genomes and favoring tumorigenesis.

Organ aging is comprehensible on a DNA methylation level and follows similar distinct patterns across human individuals. Estimators of epigenetic age, so called methylclocks, have thus been developed by linear regression analyses of CpG methylation states with chronological ages. According to each clock, the ages of sample donors and the tissue used varied, thus specifying epigenetic clocks for pediatric and/or adult tissue origin with blood- and/or tissue computational ground. Horvath for instance trained a methylclock by comparison of chronological ages with DNA methylation of various healthy tissues[18]. He deduced 353 CpG sites with a 0.99 correlation and a deviation of 3.6 years between both age parameters which makes this methylclock one of the most accurate[19]. Less accurate values were computed for heart and skeletal muscle supposedly due to epigenetically younger stem-like cells introducing noise, which is corroborated by the fact that Horvath found embryonic stem cells to have a DNA methylation age of zero. Extrapolating the findings of the Horvath methylclock on various cancer entities, a deviation between epigenetic and chronological age, called DNA methylation age acceleration, of 16 years was found[20]. Nevertheless, the presence of some mutations, like TP53, slowed down age acceleration in peripheral cancers but not in GB[20]. The tumor-intrinsic phenomena associated with aging were thus found to be inscribed on DNA methylation level but as aging is a multi-dimensional process, epigenetic aging must not be equated with cellular senescence. For leukocytes, however, there is an overlap between telomere-associated aging surrogates and immune-senescence, thus blurring clear borders between both pathways of cellular aging programs[21].

With alterations of the mitotic process and ploidity eventually also eliciting cellular epigenetic aging/senescence, the enlarged phenotype of gc as well as its mutational landscape with TP53 gene alterations and gammaH2AX accumulation, we were interested whether senescence- and/or age-associated traits were identifiable in the gcGB epigenome as opposed to its non-gc counterpart[22, 23]. We will show that on an epigenetic level, gcGB is the oldest GB variant within our cohort and is characterized by a sped-up DNAMethAgeAcc.

## Methods

### Cohort characteristics

Patients treated at the tertiary center for neurooncology of the University Hospital Frankfurt for epigenetically classified glioblastoma, IDH wild-type between 2017 and 2023 were enrolled and divided into subcohorts based on the presence (“gc-enriched GB, gcGB,” *n* = 26) or absence (“non-gc-enriched GB, non-gcGB,” *n* = 284) of giant cells, respectively. Epidemiological and clinical parameters including gender, age at diagnosis, Karnofsky performance score (KPS) prior to surgery, extend of resection and therapy as well as molecular parameters for allocation to DNA methylation classes (v11b4) and MGMT promoter methylation status were collected (Table 1). A third subcohort comprised nine adults diagnosed with diffuse gliomas and allocation of DNA methylation profiles to the methylation classes “pedHGG” and “GBM_ped” (v12.5) with classifier scores > / = 0.75. The study was approved by the local ethical committee (SNO-5-2023).

**Table 1:** Epidemiological, clinical and molecular characteristics of the main study cohort

	Gc-enriched GBM (*n* = 26)	Non-gc-enriched GBM (*n* = 284)	Fisher’s exact test
*Epidemiological and clinical parameters*			
Gender n (%)	Male 15 (58), Female 11 (42)	Male 182 (64), Female 102 (36)	0.5288
Age at diagnosis > / = 60 years n (%)			*0.0097*
Yes	10 (38.5)	186 (65.5)	
No	16 (61.5)	98 (34.5)	
Age at diagnosis median (range)	55.5 (35–83)	66 (8–86)	
KPS prior to surgery median	80	80	
Extent of resection n (%)			*0.0003*
*Total*	10 (38.5)	30 (11)	
*Non-total*	10 (38.5)	194 (68)	
*Missing data*	6 (23)	60 (21)	
Radiation + Chemotherapy n (%)			*0.0143*
*Yes*	18 (69)	156 (55)	
*No*	4 (15)	128 (45)	
*Missing data*	4 (15)	0 (0)	
*Molecular parameters*			
V11b4 HD brain tumor classifier methylation class n (%)	Glioblastoma, IDH wild-type 25 (96) Plexus tumor 1 (4)	Glioblastoma, IDH wild-type 284 (100)	*0.0068*
V11b4 HD brain tumor classifier main methylation subclass n (%)	Mesenchymal 9 (36), RTK I 8 (32), RTK II 5 (20), Midline 1 (4), MYCN 1 (4), Pediatric B (Plexus tumor) 1 (4)	Mesenchymal 113 (39.8), RTK I 66 (23.2), RTK II 92 (32.4), Midline 12 (4.2), MYCN 1 (0.4)	
MGMT promoter methylation status n (%)	Unmethylated 18 (69), Methylated 8 (31)	Unmethylated 153 (53.9), Methylated 127 (44.7), missing 4 (1.4)	0.2149

### Immunohistochemistry and scoring

By use of a microtome (Leica SM 2000R, Germany), 4-µm-thick tissue sections of formalin-fixed paraffin-embedded gcGB and non-gcGB tissue were cut and mounted on slides (Superfrost Plus, Thermo Scientific, Germany) for immunohistochemical staining following established protocols for the LEICA BOND-III automated stainer (Leica, Wetzlar, Germany). The immunohistochemical stainings were performed by use of antibodies against the following antigens: lactate dehydrogenase A (LDHA (C4B5), 1:100 dilution, Cell Signaling Technologies; Danvers, Massachusetts, USA; *n* = 21 gcGB, *n* = 21 non-gcGB), succinate dehydrogenase A (SDH-A (D6J9M), 1:200 dilution, Cell Signaling Technologies; Danvers, Massachusetts, USA; *n* = 20 gcGB, *n* = 20 non-gcGB), mitochondrial cytochrome c oxidase I (MTC-01 (1D6E1A8) 1:200 dilution, Abcam; Cambridge, UK; *n* = 21 gcGB, *n* = 21 non-gcGB), semi-purified mitochondrial preparation anti-mitochondrial antibody (MT-C02, dilution 1:100, Abcam; Cambridge, UK; *n* = 20 gcGB, *n* = 9 non-gcGB), NADH:ubiquinone oxidoreductase subunit B8 (NDUFB8 (20E9DH10C12), 1:200 dilution, Abcam; Cambridge, UK; *n* = 21 gcGB, *n* = 19 non-gcGB) and p21(Waf1/Cip1 (12D1), 1:200 dilution, Cell Signaling Technologies; Danvers, Massachusetts, USA; *n* = 18 gcGB, *n* = 21 non-gcGB). Non-gcGB case selection for immunohistochemical stainings followed age- and sex-matched to the gcGB cohort. The stained slides were examined by use of a bright-field microscope (model BX51, Olympus; Tokyo, Japan). For semi-quantitative evaluation, the Histoscore (H-Score) was applied which assesses the multiplication product of frequency (0–100%) and intensity (0 no staining, 1 week staining, 2 moderate staining, 3 strong staining) of stained cells (0–300). All stainings were evaluated by two pathologists (KJW, PC). The p53 (dilution 1:1000; Epredia; Breda, Netherlands; *n* = 25 gcGB, *n* = 243 non-gcGB) and Ki67 (MIB-1, dilution 1:200; DAKO; Glostrup, Denmark; *n* = 25 gcGB, *n* = 275 non-gcGB) immunohistochemical stainings being performed at the time of diagnosis by use of established protocols on the same device, were reassessed and documented.

### DNA methylation analysis

For DNA methylation analysis, DNA of FFPE tissue was extracted using either the Stratek Invisorb Genomic DNA Kit II (stratek molecular, Berlin, Germany) or the Maxwell RSC FFPE Plus DNA Kit (Promega, Madison, Wisconsin, USA) following the manufacturer’s protocol. DNA concentration was measured using the Qubit DNA BR Assay Kit and Qubit 3 Fluorometer device (Invitrogen, Life Technologies Corporation, Oregon, USA). DNA was processed and hybridized to the Human Methylation EPIC array beadchip (Illumina, San Diego, California, USA) following protocols provided by the manufacturer. The EPIC array beadchips were scanned by use of an iScan device (Illumina, San Diego, California, USA) and raw intensity data (idats) was generated by use of the GenomeStudio software (Illumina, San Diego, California, USA). The idats were uploaded onto the website molecularpathology.org provided by the University of Heidelberg, Germany to obtain calibrated scores for DNA methylation classes and MGMT promoter methylation status (© MolecularNeuroPathology.org 2018 and 2023). The Heidelberg brain tumor classifier versions “11b4” and “12.5” were used. The idats were analyzed by use of the R package “RnBeads” which implements quality control steps removing CpG probes of low quality, association with SNPs, sex chromosomes or potential cross-reactivity[24]. Normalization of methylation data was carried out using the “dasen” method from the R package “watermelon.” Principal component analyses from genome-wide methylation data of the whole cohort were performed by use of “RnBeads” too.

### DNA methylation-based tumor deconvolution and collection of gene-specific CpG site methylation status

Reference-free tumor deconvolution of the methylomes of all tumors was done by use of the computational algorithm “MeDeCom”[25]. This algorithm defines major patterns of data variation, called latent methylation components (LMCs), within the 5000 most variable CpGs selected for this study, and includes a biological regularization parameter lambda as well as the parameter kappa, which investigates the cross-validation error and objective value for the number of LMCs. The LMCs were standardized within the study cohort to prevent strong effects on clustering from single LMCs. All CpG sites associated with genes of interest but without association with SNPs were picked by use of the “Partek Genomics Suite” software (Partek SG Pte. Ltd., Singapore). Standardized LMCs and gene specific CpG sites were used for unsupervised hierarchical clustering according to the Ward method. Reference-based deconvolution of tumor methylomes was done using the R package “MethylCIBERSORT”[26]. By comparison with a reference matrix (provided by TRF) which provides cell type specific methylation status of selected CpG sites, the cellular composition of a tumor sample was inferred. Cell amounts of tumor cells, immune cell subsets, neurons, and glia from bulk tumor DNA methylation data were estimated following the instructions provided in the publication. Briefly, idats were imported into the R package “minfi” for quality checks, Noob normalization and acquisition of beta values. The beta value matrices were uploaded onto the CIBERSORT website and deconvoluted (provided by the Alizadeh Lab, Stanford University, USA, developed by [27]. For a second estimate of infiltrating immune cell fractions we deployed the LUMP (“leukocyte unmethylation for purity”) algorithm which provides estimates about overall leukocyte contents from bulk tumor methylomes and uses 44 CpG sites particularly hypomethylated in leukocytes[28] (implemented in RnBeads).

### Computation of epigenetic tumor ages and DNA methylation age acceleration

Different computational algorithms were used for computation of the tumors’ epigenetic ages. We computed “RnBeads” R package- and “Methylclock”-derived estimates of epigenetic ages following protocols provided in the publications[24, 29]. The methylclocks can be categorized based on training tissue and study participant ages into “pan tissue, pan age” (Horvath, Skin and Blood Horvath, RnBeads, BLUP, EN), “blood, adults” (Hannum, Levine, TL), “pan tissue, children” (Wu) and “buccal tissue, children” (PedBE). Chronological ages at the time of first diagnosis were collected from electronic patient records. DNA methylation age acceleration is calculated by subtraction of chronological from epigenetic age and displayed on an axis ranging from negative to positive values or only displaying negative values depending on the methylclock.

### Statistical analysis

Overall survival (OS) was calculated according to the Kaplan–Meier method. Log-rank- and Wilcoxon-derived p values were computed. OS in days was determined by subtraction of the date of patients’ death from the date of surgery. Patients still alive were censored at the date of their last presentation to the outpatient clinic. All statistical analyses were carried out by use of JMP17 (SAS, Cary, North Carolina, USA) or R (R Core Team, 2019). For figure design, Affinity Designer software was used (version 1.10.6.1665, Serif (Europe) Ltd, Nottingham, UK). The Pearson Chi-square test was used for categorical variables in contingency tables. For nonparametric data, Wilcoxon, Kruskal–Wallis’ and Dunn’s tests were used. A *p* value < 0.05 was considered as statistically significant. If not marked differently in the figures, the n counts for subcohort analyses comprised 26 gcGBs, 284 non-gcGBs and 9 pediatric GBs.

## Results

The core study cohort encompassed 310 epigenetically classified GBs with 26 gcGBs and 284 non-gcGBs of patients treated in the tertiary neurooncological center of the University Hospital in Frankfurt between 2018 and 2022. 64% of patients within the non-gcGB and 58% of the gcGB subcohort, respectively, were male (*p* 0.5288 Fisher’s exact test; Table 1). The percentage of patients aged 60 years or older was significantly higher among non-gcGB patients (65.5 vs. 38.5% in gcGB, *p *0.0097 Fisher’s exact test; Table 1). KPS prior to surgery did not differ significantly between the morphologic subtypes of GB (*p* 0.5 Chi^2^ test, median KPS 80, respectively). GcGB patients received total tumor resection in 38.5% of cases, whereas total tumor resection was feasible in 11% of non-gcGB patients (*p *0.0003 Fisher’s exact test; Table 1). The majority of gcGB (69%) and non-gcGB (55%) patients received radiation and chemotherapy; however, combined therapy was more often administered in patients suffering from gcGB (*p *0.0143 Fisher’s exact test; Table 1). 96% of gcGBs and 100% of non-gcGBs were allocated to the methylation class of IDH-wild-type glioblastoma by use of the Heidelberg brain tumor classifier (v11b4; *p* 0.0068 Fisher's exact test; Table 1). In detail, gcGBs were allotted to the methylation subclass “RTKI” more and “RTKII” less often than tumors of the non-gc subcohort (*p *0.0029 Pearson Chi-square test). Whereas the MGMT promoter methylation status was relatively balanced in non-gcGBs, gcGBs showed unmethylated promoters in 69% (*p* 0.2149 Fisher’s exact test; Table 1). An extended cohort comprised nine adults (mean age 64 years, range 41–73 years, 56% males) diagnosed with malignant glioma and allocation to the DNA methylation class of pediatric high-grade glioma/glioblastoma (pediatric GB; Table 2).

**Table 2:** Epidemiological and molecular characteristics of the pediatric glioblastoma study sub cohort

	Pediatric GBM (*n* = 9)
*Epidemiological and clinical parameters*	
Gender n (%)	Male 5 (56), Female 4 (44)
Age at diagnosis median (range)	64 (41–73)
*Molecular parameters*	
V12.5 HD brain tumor classifier allocation n (%)	Glioblastoma, pediatric RTK2 type, subtype B2 (22), diffuse pediatric-type high-grade glioma, RTK1 subtype, subclass A (novel) 3 (33), diffuse pediatric-type high-grade glioma, H3 wild-type and IDH wild-type, subtype A (novel) 1 (11), diffuse pediatric-type high-grade glioma, H3 wild-type and IDH wild-type, subtype B (novel) 1 (11), diffuse pediatric-type high-grade glioma, MYCN subtype 1 (11), diffuse pediatric-type high-grade glioma, RTK2 subtype, subclass B (novel) 1 (11)

### DNAMethAgeAcc is an independent prognostic biomarker in GB and gcGBs feature higher DNAMethAgeAcc independent of patient age and p53 immunohistochemical status

**Fig. 1:**
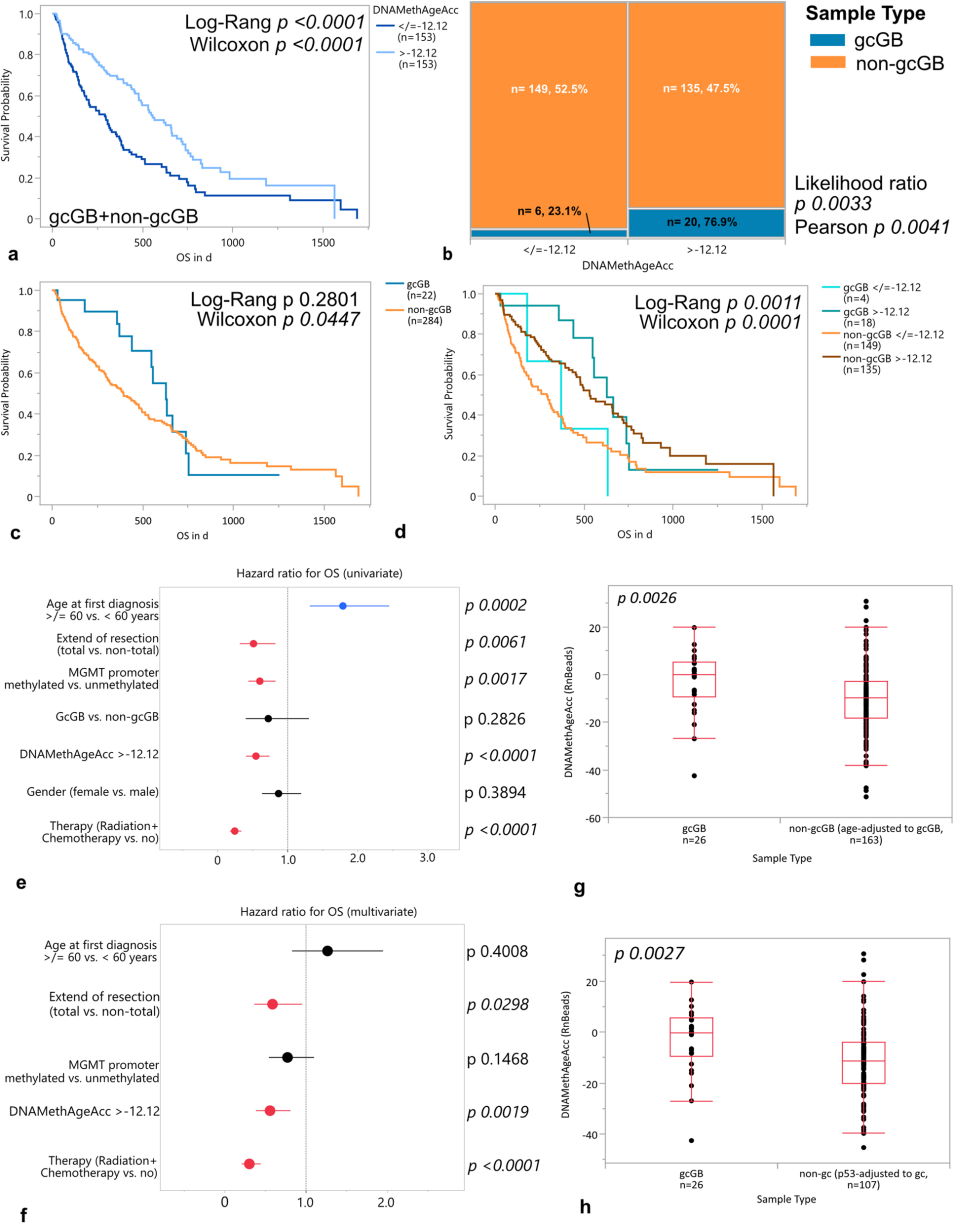
Analyses of overall survival and hazard ratios in morphologic GB variants and tumors stratified according to DNAMethAgeAcc. **a** Kaplan–Meier survival curve after cohort dichotomization to higher (> − 12.12 years) vs. lower (< / = − 12.12 years) DNAMethAgeAcc. **b** Contingency table of morphological GB variant allocation to groups with higher (> − 12.12 years) and lower (< / = − 12.12 years) DNAMethAgeAcc. **c** Kaplan–Meier survival curve for GB stratified according to morphologic variants. **d** Kaplan–Meier survival curve with sample stratification to four groups according to increased or decreased DNAMethAgeAcc for each GB morphological variant. **e** Univariate proportional hazard analysis for age at first diagnosis, extend of resection, MGMT promoter methylation status, GB morphological variant, DNAMethAgeAcc, gender, and treatment. *p* values Effect-Wald test. **f** Multivariate proportional hazard analysis for age at first diagnosis, extend of resection, MGMT promoter methylation status, DNAMethAgeAcc, and treatment. *p* values Effect-Wald test. Comparison of DNAMethAgeAcc (RnBeads-derived epigenetic ages) after adjustment of **g** chronological ages and **h** p53 immunohistochemical scores of the non-gcGB subcohort to match the gcGB subcohort. *p* values **g** and **h** Kruskal–Wallis test. Box plots in **g** and **h** with boxes representing the interquartile ranges between the 25th and 75th percentiles, the medians as line inside the box, and the whiskers the smallest and largest values, respectively

The morphologic appearance of giant cells in gcGB together with their known alterations of the TP53 pathways being involved in cellular senescence programs let us to investigate whether gcGB consists of senescent/aged tumor cells in comparison with non-gcGB. Therefore, we inferred tumor ages by use of the RnBeads pipeline and computed the DNAMethAgeAcc by subtraction of chronological patient ages from epigenetic tumor ages. Based on this approach, a median DNAMethAgeAcc of − 12.12 years was calculated for the study cohort of gcGB and non-gcGB (range − 51.32 to 30.69 years, mean − 12.44 years). After dichotomisation of the study cohort according to a median DNAMethAgeAcc of − 12.12 years, patients whose tumors showed an increased DNAMethAgeAcc > − 12.12 years survived significantly better (Fig. 1a). GcGBs were significantly more likely to allocate to the cohort moiety with increased DNAMethAgeAcc (Fig. 1b). Kaplan Meier survival analysis considering morphological variants of GB indicated a trend for better overall survival of patients with gcGB (Fig. 1c) and the combination of presence of gcs with increased DNAMethAgeAcc might point toward an added benefit for survival albeit case numbers are low (Fig. 1d). Next to treatment regimen and extend of tumor resection, DNAMethAgeAcc maintained an independent prognostic variable in uni- and multivariate analysis of hazard ratio for OS in GB (Fig. 1e, f). Increased DNAMethAgeAcc correlated negatively with chronological ages in both subcohorts; however, the increased DNAMethAgeAcc, which was found in gcGB, persisted upon harmonization of ages between non-gc and gc cases (Fig. 1g, Supplementary Fig. 1a, b). As TP53 mutations were shown to accelerate epigenetic aging in GB, we screened our cohort of gcGB and non-gcGB for an association with DNAMethAgeAcc[18]. We saw a low to moderate positive but significant correlation between p53 staining frequency and DNAMethAgeAcc (Supplementary Fig. 1c). GcGB displayed a significantly higher frequency of nuclei accumulating the cell cycle checkpoint protein p53 as assessed by immunohistochemistry (Supplementary Fig. 1d). To test whether the differences between gcGB and non-gcGB observed in this study originate from unbalanced distributions of p53 alterations, we excluded cases from the non-gcGB cohort, which lacked information about p53 IHC frequencies as well as tumors with staining frequencies lower than or equalling 25%. With median p53 staining frequencies being balanced between gcGB and non-gcGB (p53 IHC % range 0–95, median 50, *n* = 25 for gcGB, p53 IHC % range 30–100, median 50, *n* = 107 for non-gcGB; *p* 0.9227), DNAMethAgeAcc maintains significantly altered between gcGB and non-gcGB pointing toward an overly p53 alteration-independent epigenetic aging enhancer present on top in gcGB (Fig. 1h). Images of representative immunohistochemical stainings in gcGB and non-gcGB are provided in Supplementary Fig. 2.

### DNAMethAgeAcc is more advanced in gcGB than non-gcGB and pediatric GB

**Fig. 2:**
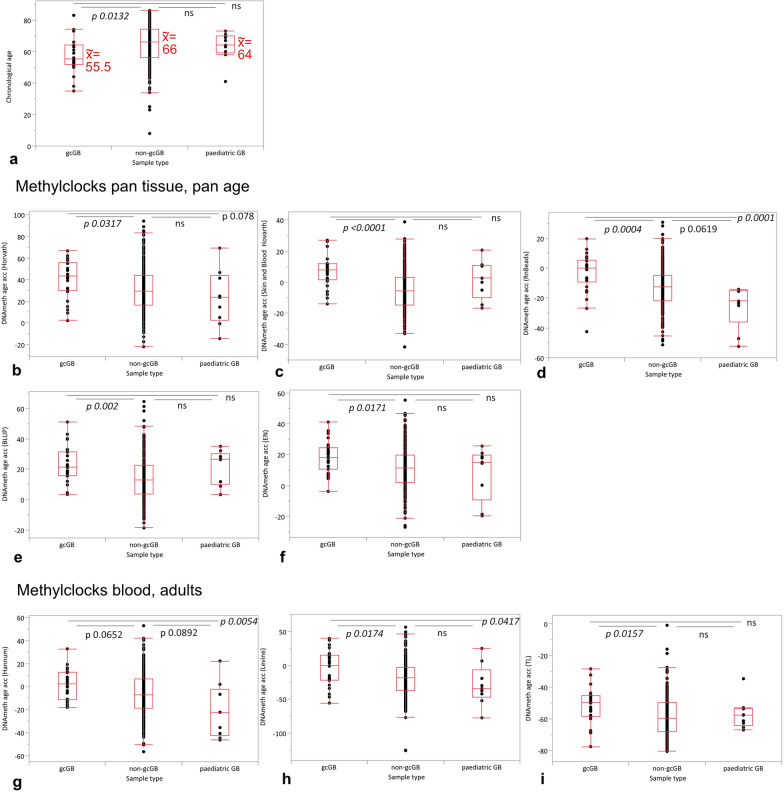
Comparison of chronological ages and DNAMethAgeAcc computed by various methylclocks in gcGB versus non-gcGB versus pediatric GB. **a** Chronological patient ages. DNAMethAgeAcc derived from methylclocks trained with pan tissue and pan ages: **b** Horvath. **c** Horvath Skin and Blood. **d** RnBeads. **e** BLUP. **f** EN. DNAMethAgeAcc derived from methylclocks trained with blood from adults: **g** Hannum. **h** Levine. **i** TL. *p* values Dunn method for nonparametric comparisons. Box plots with boxes representing the interquartile ranges between the 25th and 75th percentiles, the medians as line inside the box, and the whiskers the smallest and largest values, respectively

To understand the direction of the time ray for epigenetic aging, we also considered the molecular subclass of H3/IDH wild-type pediatric glioblastoma because of its sharp molecular distinction from adult glioblastoma probably due to an age-dependent factor inscribed into the methylome as shown by Korshunov et al.[5]. Overall, we observed no clustering of gcGB versus non-gcGB samples based on DNA methylomes in principal component analysis (Supplementary Fig. 3a). Whereas genome-wide DNA-methylation levels were significantly lower in the molecular subgroup of pediatric GB, they were balanced between gcGB and non-gcGB (Supplementary Fig. 3b). GcGB patients, who were significantly younger than non-gcGB but not pediatric GB patients in terms of chronological ages upon first diagnosis (Fig. 2a), showed a sped-up DNAMethAgeAcc in comparison with non-gcGB patients in most methylclocks, either trained on tissue and blood from adults and children (Fig. 2b–f), blood samples from adults (Fig. 2g–i) or pediatric samples only (Supplementary Fig. 3c, tissue and blood; Supplementary Fig. 3d, buccal tissue). When compared to non-gcGB, gcGB in detail showed a significantly accelerated aging of the DNA methylome in the pan tissue, pan age clocks “Horvath,” “Horvath skin and blood”, RnBeads- and Methylclock R package-incorporated algorithms “BLUP” and “EN” as well as in the “Levine” clock trained on adults’ blood samples. Significant DNAMethAgeAcc was further observed in the methylclocks “Wu” and “PedBE,” trained on pediatric specimen only (Supplementary Fig. 3c, d). The methylome of pediatric GB tumors on the contrary is significantly younger on an epigenetic level apparent in decelerated values for DNAMethAgeAcc when compared to gcGB by use of the “Horvath,” RnBeads-incorported, “Hannum,” and “Levine” methylclocks, respectively. Significant DNAMethAgeAcc differences between non-gcGB and pediatric GB were not found. Along that line, increased age acceleration was further apparent on telomere level in gcGB (Fig. 2i).

### The DNA methylome-based latent space reflects differences in GBs regarding DNAMethAgeAcc but not morphologic variants

**Fig. 3:**
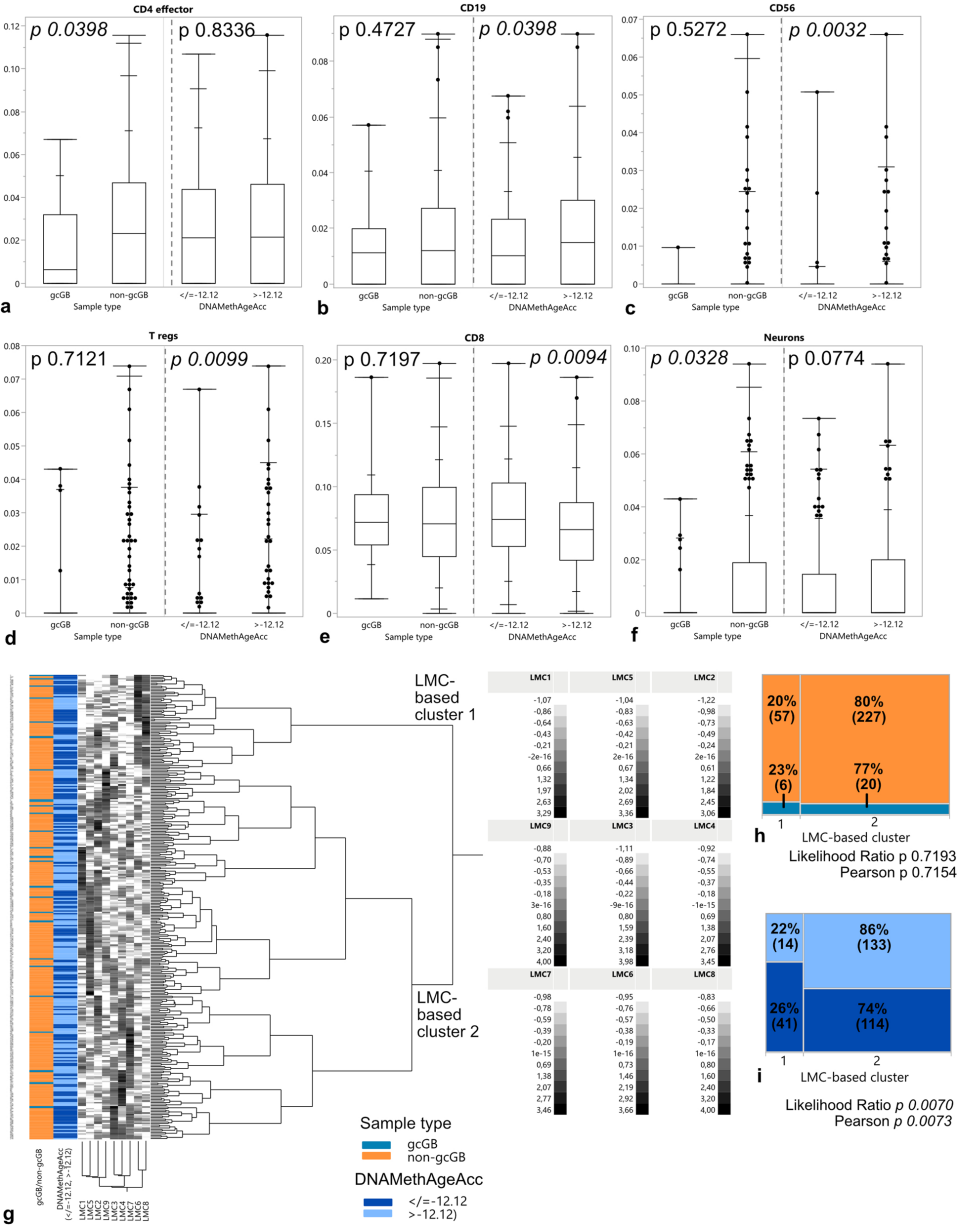
Methylome-based deconvolution of morphologic GB variants, and GBs dichotomized according to DNAMethAgeAcc. Estimates for tumor microenvironment composition, derived from MethylCIBERSORT for **a** CD4 effector cells, **b** CD19+ B cells, **c** CD56+ cells, **d** T regulatory cells, **e** CD8+ T cells and **f** Neurons. **g** Reference-free deconvolution of methylomes by computation of LMCs followed by Ward unsupervised hierarchical clustering, *k* = 9, lambda = 0.001. **h** Contingency table showing allocation of gcGB and non-gcGB samples to LMC-based cluster 1 and 2, respectively, with percentages and number of cases referring to each morphologic GB variant. **i** Contingency table showing allocation of GBs with/without DNAMethAgeAcc > − 12.12 years to LMC-based cluster 1 and 2, respectively, with percentages and number of cases referring to each cohort moiety. Box plots with boxes representing the interquartile ranges between the 25th and 75th percentiles, the medians as line inside the box, and the whiskers the smallest and largest values, respectively, and additional summary statistics with extra lines representing 90, 97.5 and 99.5 percentiles, respectively

To further search for tumor properties associated with the presence of gc and hypothetic aged/senescent microenvironments we performed methylation-based tumor deconvolution. The cellular composition of the tumor microenvironment was analyzed by use of the reference-based deconvolution algorithms LUMP and MethylCIBERSORT in gcGB versus non-gcGB as well as samples with DNAMethAgeAcc > − 12.12 versus < / = − 12.12. Considering overall leukocyte infiltrates by use of LUMP, we detected no differences between both morphologic variants and samples of different epigenetic aging (Supplementary Fig. 4a). Looking closer into immune cell subpopulations using MethylCIBERSORT, we found significantly less CD4-positive T cells in the gcGB than the non-gcGB microenvironment (Fig. 3a). The microenvironment of tumors with increased DNAMethAgeAcc on the contrary was richer in CD19-positive B cells, CD56 positive cells and T regulatory cells as well as poorer in CD8-positive T cells by use of in silico-deconvolution (Fig. 3b–e). CD14 positive cell, tumor cell and resident glia proportions did not differ between gcGB versus non-gcGB or samples with versus without increased DNAMethAgeAcc < / > − 12.12 (Supplementary Fig. 4b–d), whereas resident neurons were significantly scarcer in gcGB only (Fig. 3f). Hence, the cellular composition of gcGB and increased epigenetically aged tumor microenvironments did not show similarities in reference-based tumor deconvolution of methylomes. In a next step, we looked for differences in the latent methylation space using reference-free tumor deconvolution and computation of LMCs by use of the MeDeCom algorithm. The dataset separated into nine LMCs which led to two major subgroups after unsupervised hierarchical clustering (Fig. 3g). GcGB, however, was not distinguishable from its non-gc counterpart as it was distributed across both LMC-based clusters (Fig. 3h). Tumors with DNAMethAgeAcc > − 12.12 years, however, were significantly more frequently allocated to LMC-based cluster 2 (Fig. 3i; Fisher’s exact test *p* 0.0107).

### Epigenetic regulation of mitosis and metabolism-related genes does not differ in gcGB versus non-gcGB but GcGB shows higher protein levels of mitochondrial cytochrome c oxidase subunit II

**Fig. 4:**
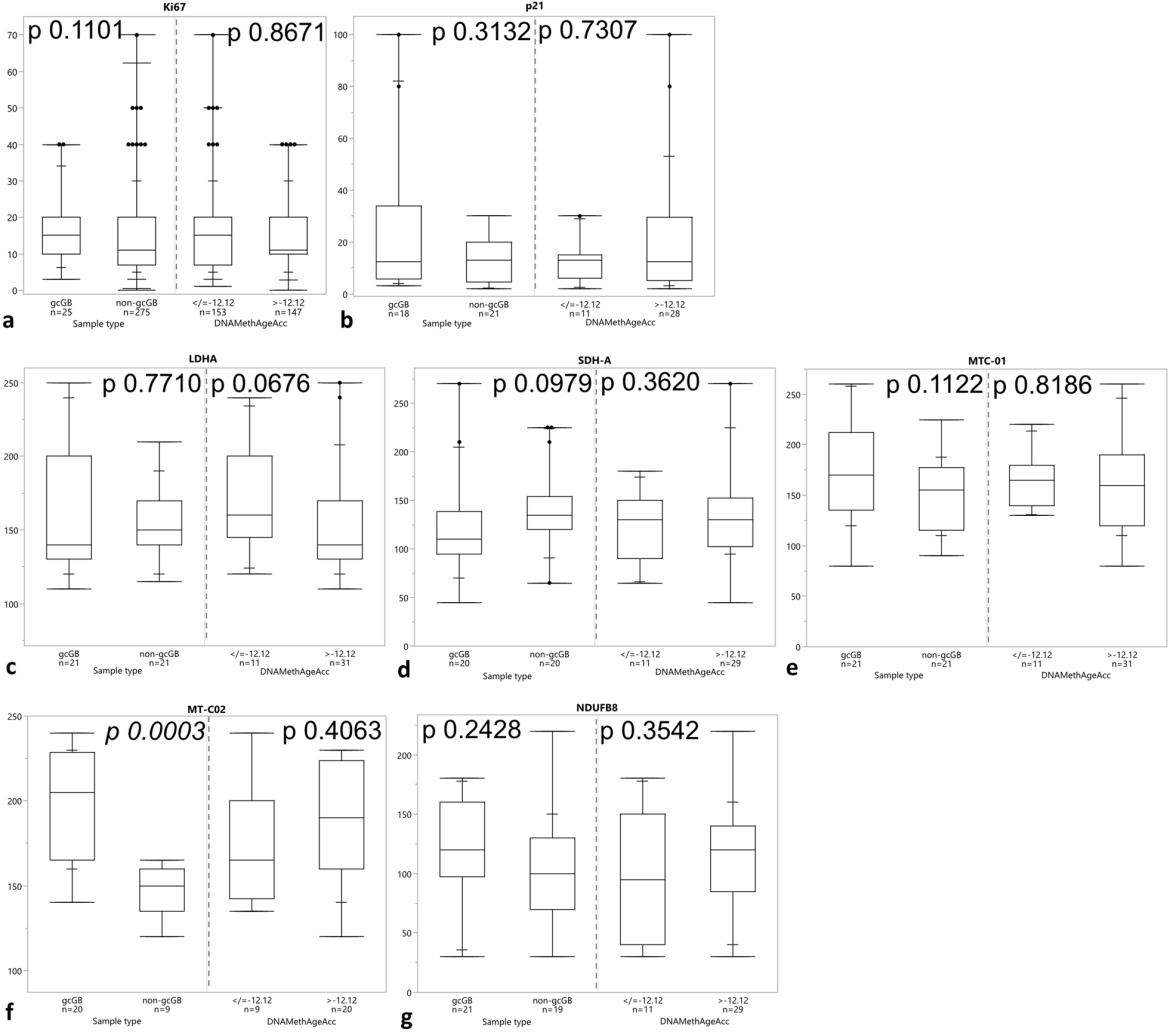
Analysis of markers for proliferative activity, cellular senescence, and key metabolic enzymes on protein level in gcGB vs. non-gcGB and GB with vs. without increased epigenetic aging. Immunohistochemical protein expression of **a** Ki-67 (estimation of positive nuclei), **b** p21, **c** LDHA, **d** SDH-A, **e** MTC-01, **f** MT-C02, **g** NDUFB8. **b**–**g** *H*-Scores. *p* values Kruskal–Wallis test. Box plots with boxes representing the interquartile ranges between the 25th and 75th percentiles, the medians as line inside the box, and the whiskers the smallest and largest values, respectively, and additional summary statistics with extra lines representing 90, 97.5 and 99.5 percentiles, respectively

As dysfunctional mitosis checkpoints have been linked with the gc phenotype, and cellular aging/senescence was characterized by an enrichment of mitochondrial mass on one hand but dysfunctional mitochondria on the other, we further set out to elucidate potential differences in epigenetic regulation of mitosis- and metabolism-related genes and key proteins[14, 17]. Therefore, CpGs associated with genes involved in mitosis were selected and subjected to hierarchical cluster analysis (Supplementary Table 1). GB with gc showed no distinct DNA methylation patterns of mitosis-related genes separating it from non-gcGB (Supplementary Fig. 5a). The frequency of Ki67-immunopositive tumor cell nuclei as estimator of proliferating activity was equitable between both morphologic variants of GB and samples dichotomised according to DNAMethAgeAcc of < / > − 12.12 years (Fig. 4a). Furthermore, p21 staining frequency in whole tumors did not differ significantly between gcGB and non-gcGB or tumors with DNAMethAgeAcc < / > − 12.12 years (Fig. 4b). We further screened the DNA methylation level and selected a panel of CpG sites associated with metabolic genes (Supplementary Table 2). Upon unsupervised hierarchical clustering we observed subgroup formations which differed according to DNA methylation patterns of metabolic genes, this, however, being independent of the presence of gc in GB, because gcGB samples did not enrich within one distinct cluster (Supplementary Fig. 5b). Next, selected metabolic proteins from the metabolic gene panel were analyzed in whole tumor sections. Lactate dehydrogenase A (LDHA) protein levels representing glycolytic activity did not differ between gcGB and non-gcGB as well as GBs with or without increased DNAMethAgeAcc (Fig. 4c). Surrogate markers for mitochondrial activity mostly also displayed no differences, respectively, but for MT-CO2, coding for the mitochondrial cytochrome c oxidase subunit II, which was significantly higher expressed on protein level in gcGB (Fig. 4d–g). Thus, we observed no differences of DNA methylation-derived control of mitosis-related or metabolic genes between gcGB and non-gcGB. On protein level nevertheless, one mitochondrial cytochrome c oxidase was significantly higher expressed in gcGB, whereas proliferative activity or senescence marker p21 immunoreactivity scores did not differ between both morphologic GB variants. Increased epigenetic aging in GB was not associated with differences in protein expression.

## Discussion

Morphologic characteristics of gcs within GB, that is, their enlarged shape, together with literature review describing increased incidence of TP53 mutations and double strand breaks in this morphologic GB phenotype, led us to the investigation of aging/senescent programs within GB. As we concentrated on DNA methylation analysis, this question was majorly addressed by use of methylclocks. We found gcGB to have an accelerated DNAMethAgeAcc in most epigenetic clocks tested compared to non-gcGB and pediatric GB.

By definition and by construction, methylclocks intend to estimate chronological ages. Cancer-related methylomes are distinct from healthy tissues’ and imply tumor-specific alterations which raises the basic question whether age estimates can be drawn from tumor tissue at all. Perez et al. looked closer into DNA methylation changes associated with aging, cancer or shared between both conditions[30]. The roughly 2000 450 k Human Methylation datasets analyses showed higher variability on a global level between healthy and malignant states than older or younger age; however, aging was rather associated with a hypermethylation in a tissue-dependent manner, whereas the cancer methylome, including also glial neoplasms, displayed both hyper- and hypomethylated CpGs without a clear trend for one DNA modification. The occurrence of shared hypermethylated sites between aging and cancer was higher than expected by chance and mainly allocated to genes related to developmental processes, but CpG hypomethylation associated with both tumor and age was majorly not found[30]. The study included brain tumors also, showing a correlation coefficient of 0.97 between chronological and epigenetic (Horvath clock-based) ages for healthy tissue which dropped to 0.33 among malignant samples. Although cancer is supposed to reprogram the epigenetic clock as hypothesized by Horvath, it remains questionable whether the findings would have been the same, when brain tumor entities had not been mixed but considered separately[18]. This is especially important if globally hypomethylated, onco-histone mutant gliomas or hypermethylated IDH mutant gliomas are considered. Within the light of gcGB and non-gcGB being indistinguishable from each other on global DNA methylation analysis as well as reference-free tumor deconvolution approaches, the differences in DNAMethAgeAcc become even more striking.

We observed epigenetic ages to be lower in non-gcGB and higher in gcGB which leads to the shift of DNAMethAgeAcc between them. In order to understand whether either the non-gcGB epigenetic reprograming leads to younger tumor cells with respect to patient age and gcGB ages non aberrantly, or gcGB reprogramming lets tumor cells age quicker, we included methylomes of the pediatric GB subtype. Taken global DNA methylation patterns into account, adult and pediatric GBs were shown to cluster separately, indicating distinct changes of the methylome which are tumor-specific and thus allow for distinction into different methylation classes[4–6, 31]. Most intriguingly, when compared with other childhood CNS tumors, pediatric GBs rather cluster with formerly PNETs and medulloblastoma than adult GBs. This rather argues for an underlying tumor age-related factor inscribed into the methylome than an introduced noise due to healthy resident cells as the pediatric GB molecular subclass also allots to adult patients, represented in our study cohort by age ranges of 41 until 73 years. By comparison of DNAMethAgeAcc between those three subgroups of IDHwt GB, we saw, that the age acceleration gap became even bigger than in the comparison between gcGB and non-gcGB arguing for a boosted aging in gcGB. This is even more striking in light of pediatric GB showing some similarities to gcGB, as it is known to harbor TP53 alterations in up to 56% and an accumulation of DNA double-strand breaks[5, 32, 33].

The algorithms underlying methylclocks weigh CpG site methylation states with relation to age[18, 24, 29]. Nevertheless, the gcGB cohort in this study was significantly younger than the non-gcGB cohort, thus supposedly introducing a systematic bias when chronological ages were subtracted from epigenetic ones to compute DNAMethAgeAcc. Analyzing gliomas of different WHO grades and IDH mutation status by use of the Horvath clock and EpiTOC, a methylclock considering a tissue’s mitotic activity, Liao et al. found tumors of chronologically older patients to harbor higher epigenetic ages[34]; our results, however, were reproducible upon age-adjustment of the non-gcGB study subcohort to fit gcGB patient ages. Further arguing against an introduced age bias through cohort selection is the fact that epigenetic programs in gcGB must have accidently and significantly altered more CpG sites interrogated by the methylclock algorithms than the one in non-gc, which is rather less likely, especially as the CpG sites called in each methylclock do largely not overlap. Nevertheless, methylclock results on glioma dataset were shown to correlate and further be able to distinguish glioma subtypes from each other pointing at differential signatures of epigenetic aging[34]. GcGBs might therefore constitute a subgroup within GB with a distinct aging signature on an epigenetic level. In GB, giant cell enrichment was described to co-occur with defective DNA mismatch repair [10, 12]. Although the accumulation of somatic mutations in DNA mismatch repair deficiency was not strictly linked to giant cell accumulation in GB, and hypermutation with > 10 mutations/Mb found in up to 41% of gcGBs was independent of gc proportions within GB, this co-occurrence questions gcGb-specific genomic alterations potentially promoting epigenetic aging [12, 35]. In addition to mutations of genes related with mismatch repair deficiency gcGB was found to harbor higher mutational frequencies of *RB1, NF1, TP53 and ATRX* genes in comparison with conventional glioblastoma, whereas *EGFR* alteration occurred less frequent [12]. Beyond the Rb/p16inka and p53/p21 pathways hijacked in both neoplasia and senescence, the overlap between altered genes, typically associated with senescence and the gcGB-specific mutational landscape altogether is rather small [17, 23, 36]. Still, a one-to-one translation from cellular signaling patterns related to senescence to mechanisms fostering epigenetic aging is not feasible. With the methylclock’s ticking being related to physiological organ aging and cellular differentiation which per se do not succumb to patterns of genomic mutations but follow a trend of leveling DNA hyper- and hypomethylation, the association of epigenetic aging and genomic alterations is unassertive [18, 19]. There are, however, some genomic alterations which have been specifically linked to epigenetic aging. In breast carcinoma, steroid receptor mutations sped-up epigenetic aging as did TP53 mutations in many cancer entities but GB, where the latter were shown to have the contrary effect [18]. Along that line, a negative correlation between the mutational count and epigenetic age acceleration was described, which adds to the complexity of the gc-enriched GB phenotype [18]. Nevertheless, the lack of information on somatic mutational counts along with defects of DNA repair mechanisms especially in the gcGB subcohort are limitations of this study. In addition, a site-specific quantification of changes of methylation in gcGB versus non-gcGB with respect to CpG sites called by the methylclocks as well as a correlative analysis between cohorts targeting senescence-associated chromatin remodeling stretches beyond the scope of this study.

When on a cellular level aging is considered as gradual loss of function, it is tempting to speculate that in the case of gcGB this might translate into more inert, less invasive tumor cells[23]. In fact, many studies described gcGB to invade the surrounding brain parenchyma only minimally and although tumor size and location were not found to differ significantly from non-gcGB, gcGB patients received more extensive resections, which might indirectly also point toward a less invasive behavior[7, 10]. Corroborating this, upon reference-based deconvolution of methylomes, we found significantly lower amounts of neurons within the gcGB tumor bulks than that of the non-gcGB. There are some indications in literature that multinucleation blocks cell proliferation. Fujita et al. for example assign a more passive role to cells with a multinucleated than a mononucleated phenotype in GB[14]. Differential staining patterns of phosphorylation-specific antibodies to track tumor cell cycle stages showed that multinucleated cells got stuck in early mitotic phases[14, 37]. Another study about the effects of multinucleation on cell cycle progression showed a disruption before entering S phase. Hart et al. observed multinucleated cells to arrest in G1 phase even in a p53-compromised setting which was associated with p21 accumulation and absence of PCNA foci as indicators of S phase, thereby pointing toward the initiation of cellular stress programs which prevented the resumption of cell cycling[13]. Most interestingly, those cells were still viable and showed transcription in regions of the genome, not subjected to severe double strand breaks which is reminiscent of the senescent cell state with continued paracrine and transcriptional activity but exit from cell cycling[13, 38, 39]. Furthermore, we did not detect different staining frequencies of p21 when considering whole tumor sections in the study subcohorts. Besides the fact that cellular senescence and epigenetic aging must not be equaled, increased nuclear accumulation of p21 might not be a very reliable marker of senescent cells, as it might be difficult to detect by use of immunohistochemistry because of its heterogenous expression through compartments within the multinucleated cells and its verifiability also in quiescent cells[13, 17]. Stainings with the senescence marker beta-Galaktosidase would be an important add-on for future studies on gcGB to improve our understanding of quiescent versus senescence states in these cells.

A cell can enter the senescence state upon any oncogenic stress in order to prevent malignant degeneration. The consequent growth arrest is a point of no return unless its gatekeepers, and the p53/p21 and Rb/CDKN2A pathways are enabled. An activated senescence program elicits changes on chromatin level, known as senescence-associated heterochromatin foci, but stretched also beyond the single cell level[40]. Senescent cells with impaired genomes or epigenomes have been shown to secret cytokines, growth factors, proteases, and chemokines, summarized under the term SASP which acts on the microenvironmental level and is supposed to also hold back cancer establishment[23]. In our reference- and methylation-based deconvolution of gcGB versus non-gcGB tumor bulk we found lower proportions of CD4+ T cells in the gcGB microenvironment. This is counterintuitive on one hand, because SASP can promote leukocyte infiltration thereby leading to an apathogenic, low-level chronic inflammation, but on the other hand might reflect an age-dependent drop in the overall CD4+ T cell population in addition to increasing levels of IL-6 associated with aging and their negative effect on CD4-mediated antitumor response within an assumptive aged/senescent gcGB microenvironment[41–43]. Surprisingly, the cellular composition of the gcGB microenvironment did not overlap with the one in silico deconvolution approaches computed for tumors with increased DNAMethAgeAcc. This might be associated with the samples’ allocation to DNA methylation subclasses as these were repeatedly found to show different levels of immune cell infiltration, especially for T cells[44]. In line with Jeanmougin et al. delineating a beneficial prognostic impact when higher proportions of immune cells are present within the GB microenvironment, our tumor deconvolution analyses point toward more infiltration of B cells, NK cells and T regulatory cells into the microenvironment of the more favorable tumors with increased DNAMethAgeAcc[45]; Lower proportions of CD8-positive T cells in the latter are nevertheless unintelligible, but might argue for the importance of functional status of T cell subsets to exert their tumor-suppressive role thereby questioning the accuracy of reference-based deconvolution in terms of immune cell subtype distinction[46]. Adding to the need of approaches with strong discriminatory power between cell types, the accuracy and reliability of in silico-deconvolution by use of DNA methylation were shown to largely depend on concordance between the samples and the reference matrix used for deconvolution both in terms of tissue type and analysis technique as well as bioinformatical pipelines to assure overall high site-specific and genome-wide assay quality [47]. Future studies with multimodal approaches to investigate the microenvironment of epigenetically more or less aged GBs are therefore required for validation and represent a limitation of this study.

As dysfunctional mitochondria count for a hallmark of aged/senescent cells, we assessed DNA methylation patterns of nuclear coding mitochondrial genes together with genes associated with metabolism in general[17]. Our data contradict differential metabolic, epigenetically controlled programs between gcGB and non-gcGB. On one hand, our findings on protein level were not consistent as we admittedly observed increased staining frequency and intensity against MT-CO2 but could not corroborate a supposed accumulation of mitochondria indicative of a senescent phenotype by use of other antibodies. On the other hand, increased MT-C02 scores were characteristic of gcGBs only and not found in prognostically more beneficial tumors with increased DNAMethAgeAcc which might subtly purport the involvement of mitochondrial processes in the gcGB phenotype. Other techniques than immunohistochemistry should be considered to answer these questions in gcGB, because it neither captures structural changes of senescent mitochondria, nor their functionality or accurate number[17, 48, 49].

The gcGB phenotype consolidates nevertheless some cellular and molecular characteristics which support the hypothesis of gcGB being the “older” variant among IDHwt GB. Besides its tumor cells’ morphology, it was shown to accumulate DNA double strand breaks, involves lower invasion capability and harbors increased DNAMethAgeAcc metrics which all renders an activation of senescence programs in gcGB conceivable[10]. Yet, this hypothesis still awaits its final acceptance or rejection which can only be achieved by careful implementation of multilayered data from the ex vivo analysis of senescence-associated beta-Galactosidase staining patterns, cell cycling and proliferation parameters and structural as well as molecular properties of the nucleus bearing in mind that quiescent cells might cause false-positive results[17, 48].

Together with the fact that we saw boosted DNAMethAgeAcc in gcGB, increased DNAMethAgeAcc was significantly and independently associated with better patient survival. In addition to serving as prognostic biomarker, an increased DNAMethAgeAcc might point at distinct cellular programs worth enhancing in malignant gliomas. This is in line with the comprehensive analysis of IDH wild-type GB datasets by Bady et al., who proved the prognostic importance of a sped-up DNAMethAgeAcc by use of HumanMethylation 450 k data and the Horvath methylclock[50]. In this study, increased age acceleration was associated with the RTK II molecular subtype of GB rather than with RTK I or mesenchymal subclasses; while it was additionally shown to be independent of copy number variations and tumor purity methylclock-associated CpG sites overlapped significantly with those called for assignment of molecular subclasses[50]. These findings highlight the additive value of tumor-intrinsic epigenetic aging as clinical biomarker and argue for further studies to explore these mechanisms’ overlap with cellular senescence pathways. Whether those pathways may be targeted therapeutically to guide malignant glioma toward a biological dead end and giant cells might foster that development in GB warrants future examination.

## Conclusions

The presence of enlarged, p53 accumulating giant cells in GB does not alter the global DNA methylome when compared to non-gcGB but elicits an up-speeding DNAMethAgeAcc. This boost in epigenetic age acceleration became even more apparent in comparison with pediatric GB and repositions gcGB as the epigenetically oldest tumor variant in line. Across the whole study cohort increased DNAMethAgeAcc was tantamount to a significant survival benefit for patients with GB highlighting its biomarker capability. Tumors with prognostically favorable increased DNAMethAgeAcc were identifiable in the latent methylome space by use of reference-free tumor deconvolution. Whether DNAMethAgeAcc associates with cellular senescence/aging programs and whether these would be exploitable in future GB treatment warrants further studies.

**Supplementary Figure 1: a)** Correlation of DNAMethAgeAcc computed with the RnBeads pipeline with chronological age in non-gcGB patients. **b)** Correlation of DNAMethAgeAcc computed with the RnBeads pipeline with chronological age in gcGB patients. **c)** Correlation analysis of DNAMethAgeAcc computed with RnBeads-derived estimates of epigenetic ages in gcGB and non-gcGB with respect to frequency of p53 immunoreactive tumor nuclei. **d)** Immunohistochemical protein expression levels of p53 in gcGB vs. non-gcGB. Box plots with boxes representing the interquartile ranges between the 25th and 75th percentiles, the medians as line inside the box, and the whiskers the smallest and largest values, respectively. **Supplementary Figure 2:** H&E and immunohistochemical staining pattern in gcGB (a)-i)) and non-gcGB (j)-r)). H&E a), j) with giant cells marked by arrow in a). p53 b), k). Ki67 c), l). MT-C02 d), m). MTC-01 e), n). LDHA f), o). SDH-A g), p). NDUFB8 h), q). p21 i), r). **Supplementary Figure 3: a)** Principal component analysis of global DNA methylomes of gcGB and non-gcGB. **b)** Genome-wide DNA methylation analysis between gcGB, non-gcGB and pediatric GB. P values Dunn method for nonparametric comparisons. Comparison of DNAMethAgeAcc between gcGB, non-gcGB and pediatric GB computed by methylclocks trained on **c)** pan tissue of children (“Wu”), **d)** buccal tissue from children (“PedBE”) P values Dunn method for nonparametric comparisons, respectively. Box plots in b), c) and d) with boxes representing the interquartile ranges between the 25th and 75th percentiles, the medians as line inside the box, and the whiskers the smallest and largest values, respectively. **Supplementary Figure 4: a)** LUMP algorithm results for overall leukocyte infiltration, based on DNA methylation data, in gcGB vs. non-gcGB, and GB with DNAMethAgeAcc </= vs. >-12.12 years. Estimates of cellular composition of gcGB, non-gcGB, GB </= and >-12.12 years DNAMethAgeAcc by use of MethylCIBERSORT for **b)** CD14+ cells, **c)** tumor cells, **d)** glia. P values Kruskal-Wallis test. Box plots with boxes representing the interquartile ranges between the 25th and 75th percentiles, the medians as line inside the box, and the whiskers the smallest and largest values, respectively and additional summary statistics with extra lines representing 90, 97.5 and 99.5 percentiles, respectively. **Supplementary Figure 5: a)** Analysis of methylation status of mitosis-associated genes, unsupervised hierarchical ward clustering in morphologic GB variants. **b)** Unsupervised hierarchical ward clustering of methylation status of CpG sites associated with metabolic genes in morphologic GB variants. **Supplementary Table 1:** List of mitosis-related genes, selected for comparison between gcGB and non-gcGB by use of all annotated CpG sites present on the Human Methylation EPIC array. **Supplementary Table 2:** List of metabolic genes, selected for comparison between gcGB and non-gcGB by use of all annotated CpG sites present on the Human Methylation EPIC array.

## Supplementary Information

Additional file 1

Additional file 2

Additional file 3

Additional file4 (JPG 393 KB)

Additional file5 (JPG 3123 KB)

Additional file6 (DOCX 12 KB)

Additional file7 (DOCX 12 KB)

Additional file8 (XLSX 165 KB)

## Data Availability

All data generated or analyzed during this study are included in this published article and its supplementary information files.

## Abbreviations

CNS: Central nervous system
DNAMethAgeAcc: DNA methylation age acceleration
GB: Glioblastoma
Gc: Giant cell
IDH: Isocitrate dehydrogenase
KPS: Karnofsky performance score
LDHA: Lactate dehydrogenase A
LMC: Latent methylation component
MTC-01: Mitochondrially encoded cytochrome C oxidase I
MT-C02: Mitochondrial cytochrome C oxidase subunit II
NDUFB8: NADH dehydrogenase [ubiquinone] 1 beta subcomplex subunit 8, mitochondrial
Non-gc: Non-giant cell
Pediatric GB: Pediatric-type high-grade glioma/glioblastoma
SDH-A: Succinate dehydrogenase A
TME: Tumor microenvironment
WHO: World Health Organization
