# Supplementary figures and images for: DNA methylation-based analysis reveals accelerated epigenetic aging in giant cell-enriched adult-type glioblastoma
Source: Clin Epigenetics. 2024 Dec 11;16:179. doi: 10.1186/s13148-024-01793-w (PMC11636044; doi:10.1186/s13148-024-01793-w)

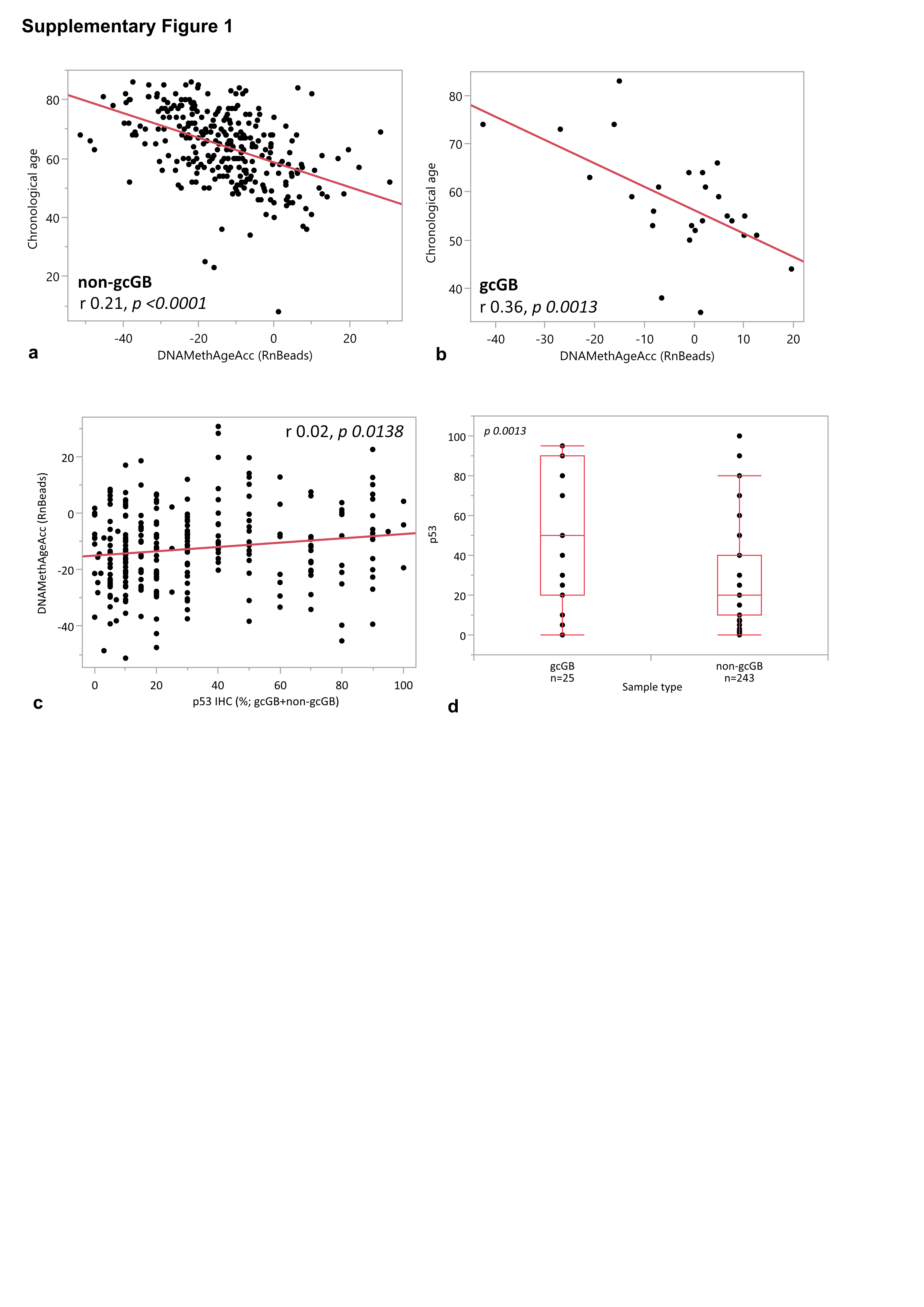

Supplement: Supplementary file 1 — Additional file 1 [file 13148_2024_1793_MOESM1_ESM.jpg]

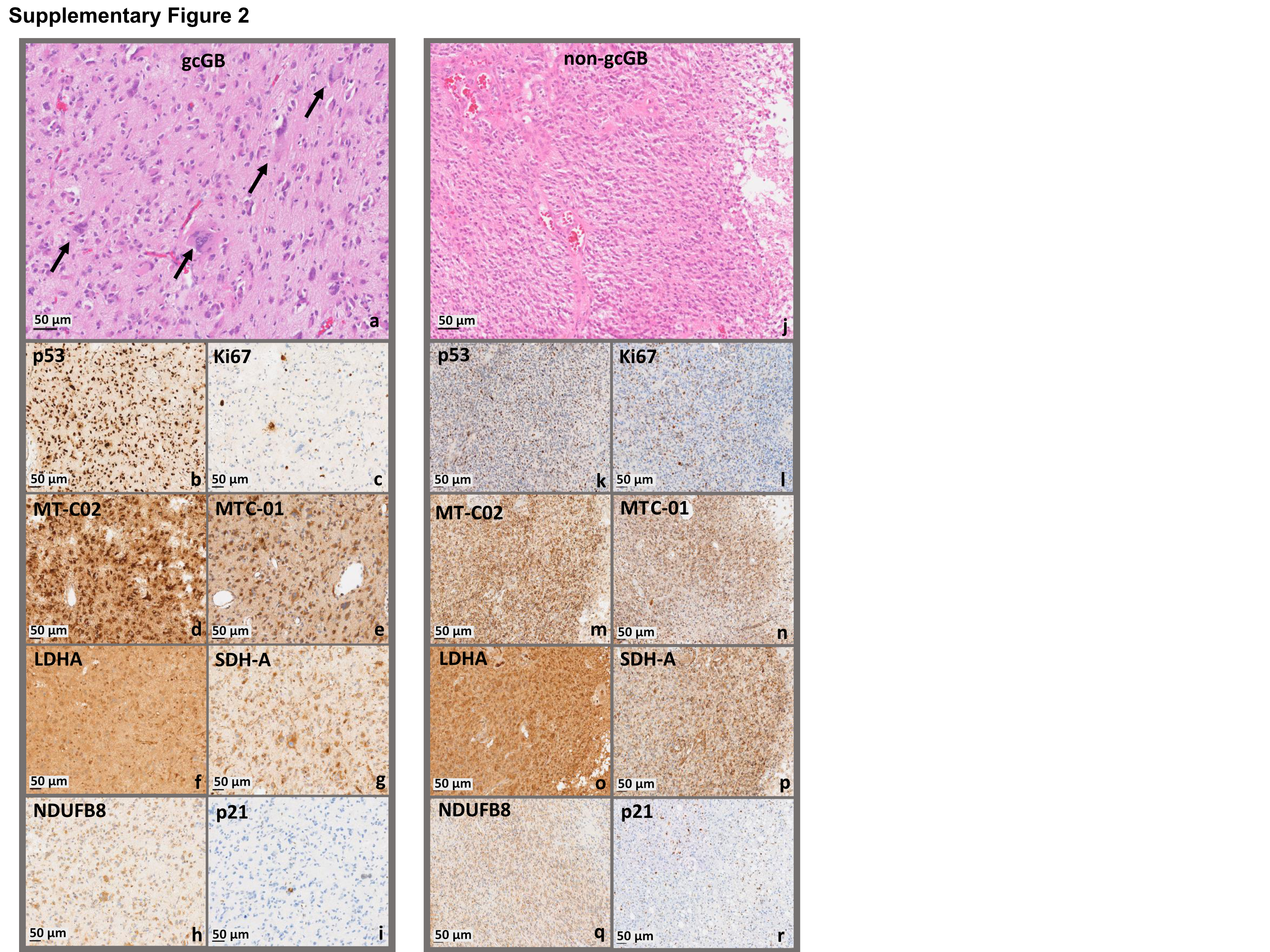

Supplement: Supplementary file 2 — Additional file 2 [file 13148_2024_1793_MOESM2_ESM.jpg]

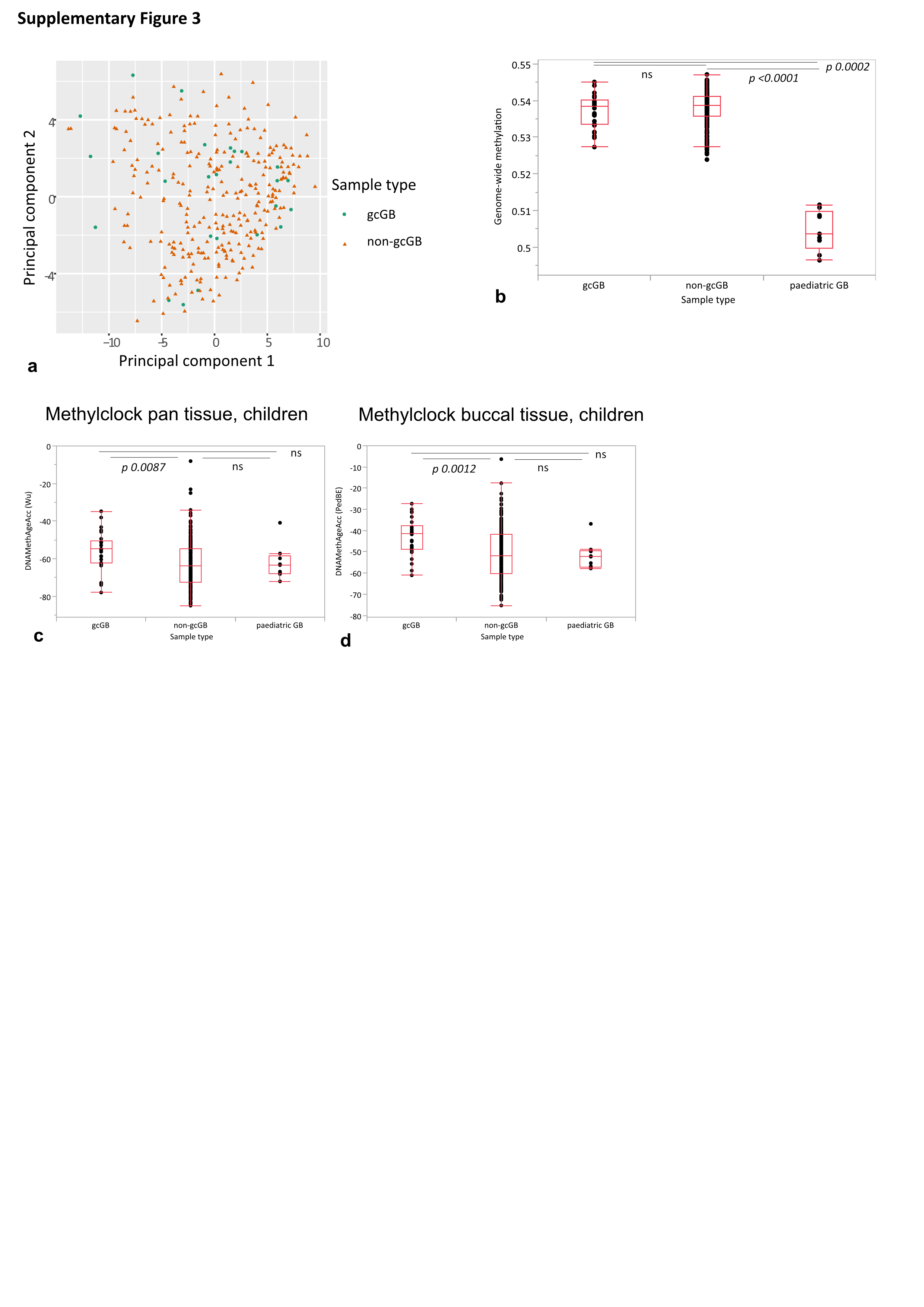

Supplement: Supplementary file 3 — Additional file 3 [file 13148_2024_1793_MOESM3_ESM.jpg]

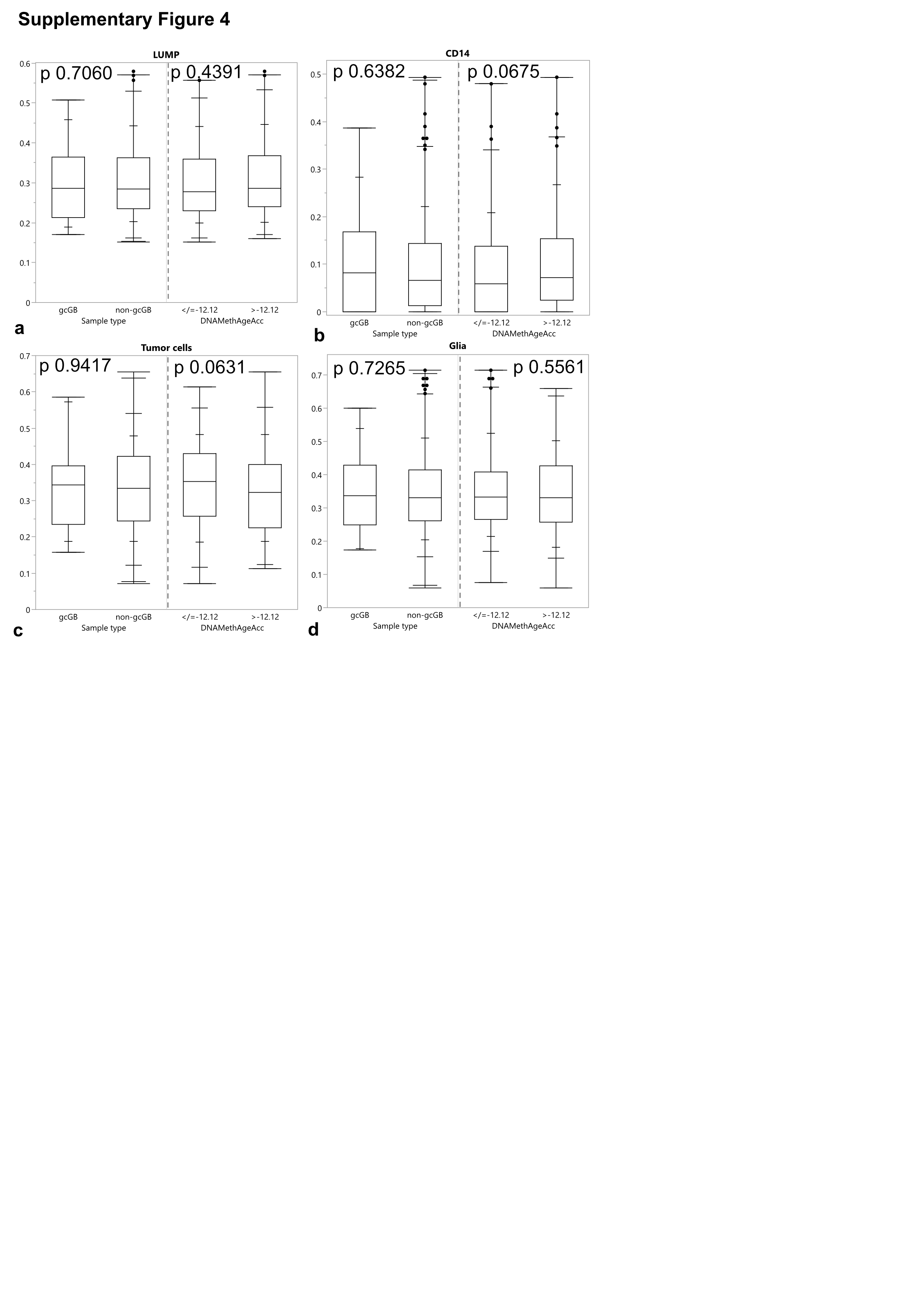

Supplement: Supplementary file 4 — Additional file4 (JPG 393 KB) [file 13148_2024_1793_MOESM4_ESM.jpg]

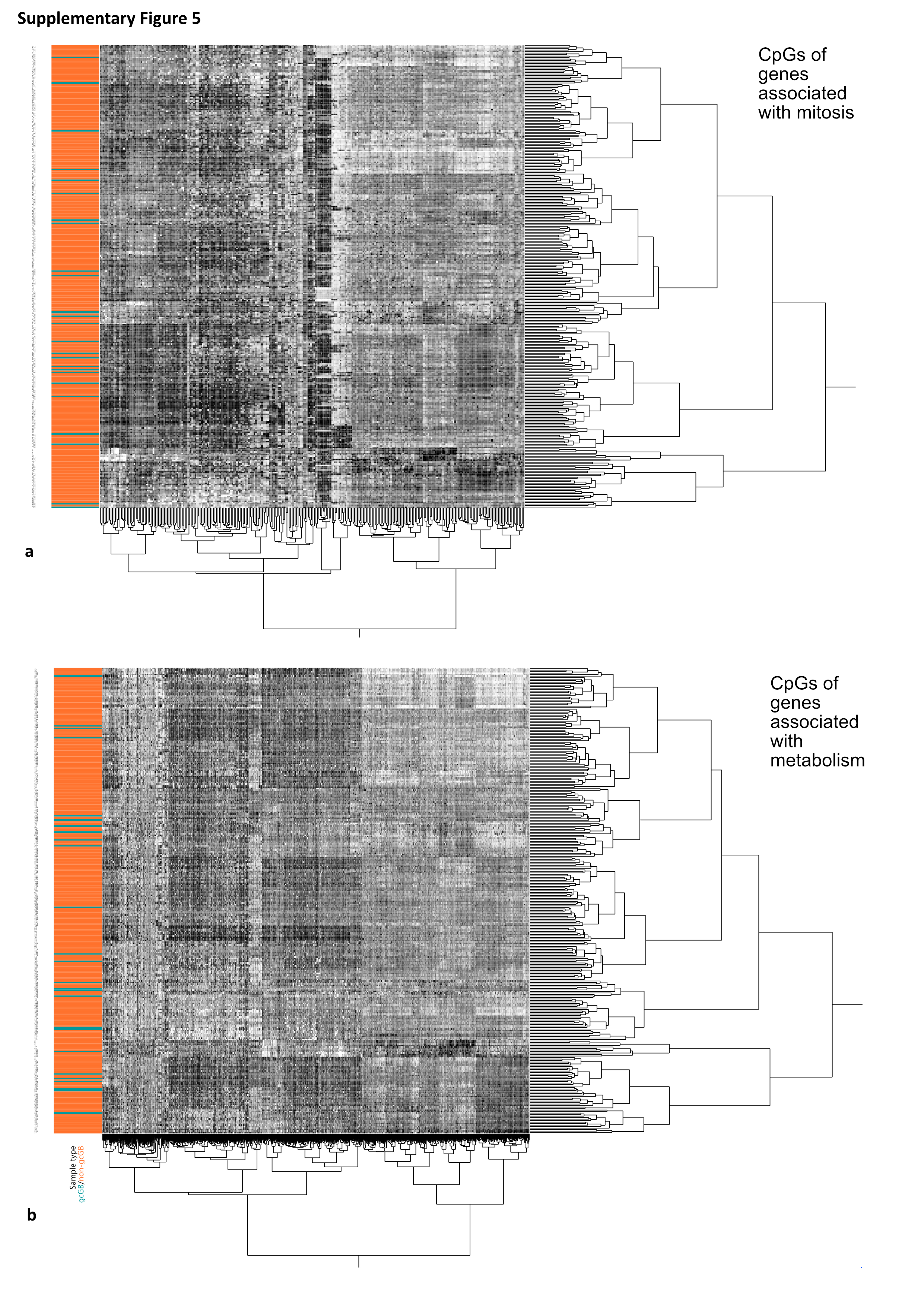

Supplement: Supplementary file 5 — Additional file5 (JPG 3123 KB) [file 13148_2024_1793_MOESM5_ESM.jpg]
