# Supplementary material for: DNA methylation-based analysis reveals accelerated epigenetic aging in giant cell-enriched adult-type glioblastoma
Source: Clin Epigenetics. 2024 Dec 11;16:179. doi: 10.1186/s13148-024-01793-w (PMC11636044; doi:10.1186/s13148-024-01793-w)
Supplement: Supplementary file 6 — Additional file6 (DOCX 12 KB) [file 13148_2024_1793_MOESM6_ESM.docx]

List of mitosis-related genes, selected for comparison between gcGBM and non-gcGBM by use of all annotated CpG sites present on the HumanMethylation EPIC array:

AURKA, AURKB, AURKC, CCNB1, CCNB2, CDC20, CDC25C, CDK1, MLH1, PLK1, PLK2, PLK3, PLK4, PLK5, TERF1, TERF2
