# Supplementary material for: DNA methylation-based analysis reveals accelerated epigenetic aging in giant cell-enriched adult-type glioblastoma
Source: Clin Epigenetics. 2024 Dec 11;16:179. doi: 10.1186/s13148-024-01793-w (PMC11636044; doi:10.1186/s13148-024-01793-w)
Supplement: Supplementary file 7 — Additional file7 (DOCX 12 KB) [file 13148_2024_1793_MOESM7_ESM.docx]

List of metabolic genes, selected for comparison between gcGBM and non-gcGBM by use of all annotated CpG sites present on the HumanMethylation EPIC array:

LDHA, SDHA, NDUFB8, ALDOA, ALDOB, ALDOC, ATP5A1, ATP5B, ATP5C1, ATP5D, ATP5E, ATP5F1, ATP5G1, ATP5G2, ATP5G3, ATP5H, ATP5I, ATP5J, ATP5L, ATP5O, ATP5S, ATPIF1, COX4I1, COX5A, COX5B, COX6A1, COX6A2, COX6B1, COX6C, COX7A2, COX7A2L, COX7B2, COX7C, COX8A, CS, DLD, DLST, ENO1, ENO2, ENO3, ESRRA, FH, GAPDH, GPI, HK1, HK2, HK3, IDH2, IDH3A, IDH3B, LDHB, LDHC, MDH2, MPC1, NDUFA3, NDUFA10, NDUFA12, NDUFA4, NDUFA5, NDUFA6, NDUFA7, NDUFA9, NDUFAB1, NDUFB10, NDUFB2, NDUFB3, NDUFB4, NDUFB5, NDUFB6, NDUFB7, NDUFB8, NDUFB9, NDUFC1, NDUFC2, NDUFS1, NDUFS2, NDUFS3, NDUFS4, NDUFS5, NDUFS6, NDUFS8, NDUFV1, NDUFV2, NDUFV3, NRF1, PDHA2, PFKP, PGAM1, PGAM2, PGK2, PKM2, POLRMT, PPRC1, SDHB, SDHC, SDHD, SUCLG1, SUCLG2, TACO1, TEFM, TFAM, TFB1M, TFB2M, UCRC, UQCR, UQCRB, UQCRC1, UQCRC2, UQCRFS1, UQCRH
